# Feeding the Sick

**Published:** 1880-08

**Authors:** 


### FEEDING THE SICK.

An intelligent officer in the army remarked one day that he had long observed that a larger number of men who were sent home to their friends sick, died than of those who remained in the army. On being asked his opinion as to what was the reason of such a result he said, " Because their friends feed them up so." The answer was undoubtedly the true one. As in health we eat heartily, it seems to be concluded that if a sick person can be made to eat heartily he will get well right away, and to induce them thus to eat, all the delicacies and tempting things that can be thought of are crowded in on the poor laboring stomach, internal fever is excited, no nourishment is drawn out of it, and the invalid consumes away by internal fires

We constantly read of persons horribly wounded in war or otherwise injured, who, unable to obtain human aid, are exposed to rain and night air and other inclemencies and would have starved, but for the roots and berries they crawled about to procure, and yet survived. It is a rule, with very few exceptions, that the sick should not have their appetites tempted; that they should wait until they felt as if plain bread and butter would taste good to them, and even then, not eat at less than five hours interval.

Tho stomach is weak like every other part of the body, and to put upon it a task which its instincts do not seek, is unwise and illogical in the highest degree. You can't make a sick pig eat, while man, a bigger pig, "forces" the food upon himself when he has not the slightest inclination and even takes measures to create the inclination. Nor bird, nor beast, nor creeping thing will eat when sick, but man, the biggest brute of all, will.

One plain wholesome dish for an invalid is prepared thus—

Take some common Indian corn, roast it as you would coffee, grind it in a coffee mill and make it, in the usual way, into a mush or gruel, or make it into thin cakes nicely browned and eat either cold or hot, alone or with sugar or salt or syrup or butter, in whatever way the stomach will receive it most kindly and retain it. This parched corn meal or parched rice, boiled in sweet milk, is one of the best non medicinal remedies known for the relief of diarrhœa or even dysentery, if the patient will remain quietly in bed for a day or two. This is especially valuable for children suffering with bowel complaint.

Another excellent article of food, wholesome and nutritious, is common wheat cleaned and washed, then let it be soaked in warm water and when the grains have softened and swollen, boil slowly until soft enough to be eaten with milk or sugar or syrup, according to the taste; it may be salted a little; if any is left over it may be cut in thin slices and then fried like mush.

It is well known among physiologists that the teeth and bones are durable and strong in proportion as they contain one of the chemical constituents of lime, and that the food which contains these constituents in large quantities is best adapted to the formation of good teeth and strong limbs. In the item of bread, used in every family, a striking fact is exhibited: in 500 pounds of the finest flour for table use there are thirty pounds of these bone-forming elements; in an equal amount of bread made of wheat, or prepared as above, there are eighty-five pounds of the bone and tooth-forming principles, hence it is not to be wondered at, that the Scotch are the thriftiest and hardiest race in the world, for they luxuriate on their dearly beloved oat-meal gruel, bread and cakes three times a day. The whole grain of Indian corn or wheat prepared as recomme d-ed does not fatten as much as fine flour, the latter having t rice the amount of fat-forming principle; but fat is not strength; it does not give endurance, toughness, hardness, capability of work; the whole grain of the Indian, wheat, rye, oats, does, and from five to fifteen, children should be compelled to make one daily meal, wholly, of one of these grains, prepar d as above.

